# The Role of VATS in Lung Cancer Surgery: Current Status and Prospects for Development

**DOI:** 10.1155/2015/938430

**Published:** 2015-07-29

**Authors:** Dariusz Dziedzic, Tadeusz Orlowski

**Affiliations:** Department of Thoracic Surgery, National Research Institute of Chest Diseases, Warsaw, Poland

## Abstract

Since the introduction of anatomic lung resection by video-assisted thoracoscopic surgery (VATS) 20 years ago, VATS has experienced major advances in both equipment and technique, introducing a technical challenge in the surgical treatment of both benign and malignant lung disease. The demonstrated safety, decreased morbidity, and equivalent efficacy of this minimally invasive technique have led to the acceptance of VATS as a standard surgical modality for early-stage lung cancer and increasing application to more advanced disease. Formerly there was much debate about the feasibility of the technique in cancer surgery and proper lymph node handling. Although there is a lack of proper randomized studies, it is now generally accepted that the outcome of a VATS procedure is at least not inferior to a resection via a traditional thoracotomy.

## 1. Introduction

The concept of thoracoscopy was first described in 1910 by Jacobaeus, an internist, for the management of pleural effusion with a urological cystoscope [1]. A simple cystoscope was used, with a rigid working channel and an illumination source at its end, with which the pleural space could be directly visualised. The procedure was performed under local anaesthesia. In the years to follow, thoracoscopy has become a common therapeutic procedure for the lysis of pleural pulmonary adhesions caused by tuberculosis during collapse therapy to create a pneumothorax. The number of thoracoscopy procedures in tuberculosis has decreased with the introduction of streptomycin in 1945 as the first effective antituberculosis chemotherapy. In the following years, thoracoscopy has been mainly used for diagnosis. New methods of diagnostic imaging utilizing improved light delivery system and the newly developed diagnostic imaging devices combining a hollow tube and a camera to transmit image to a screen to capture images of the operating field have prompted the development of thoracoscopic techniques. The notion of video-assisted thoracoscopic surgery (VATS) has emerged. VATS continued to evolve in close connection with technical advancements in imaging techniques and the refinement of surgical instruments, which made room for new indications for VATS. The introduction of a “cold” halogen light source was another major step in enhancing the visualization of anatomical structures. This was a major development in VATS since the blood in the operation field would absorb up to 50% of the light [2]. Angled lens were another improvement that helped visualize anatomic structures that are difficult to access. Another milestone in VATS development was the introduction of endostaplers. Presenting endostaplers guided by VATS opened the way for effective and safe lung parenchyma through a tiny incision. From then on, video-assisted thoracoscopy (VATS) has been gaining popularity in thoracic surgery not only in diagnosis, but also in the management of pneumothorax, resection of small pulmonary nodules, or in the treatment of thoracic injuries. This is how VATS (video-assisted thoracic surgery) emerged and evolved, which many surgeons believe is clearly distinct from traditional thoracoscopy. The first ever large-scale symposium dedicated to VATS was staged by the Society of Thoracic Surgeons in San Antonio, Texas, in January 1992 [3]. Also in 1992, the first anatomic resection was performed with VATS [4]. The main idea behind popularization of VATS was to significantly reduce procedure-related injury and complications that frequently accompanied traditional open thoracotomies.

## 2. Definition

VATS has no universally recognised definition. In general, a variety of different VATS techniques is used. The procedure of lung resection in cancer patients is referred to as VATS lobectomy. The essential assumptions of VATS lobectomy are to make 1.5–2 cm access incisions for 2 to 4 thoracoscopic ports, and 2–6 cm access incisions in the anterior part of the thorax for a minithoracotomy (utility incision). Number of ports (with 1, 2, or 3) through which surgical instruments are inserted varies and depends on the experience of the surgeon. The VATS approach does not require any rib spreading, resulting in a less invasive injury in the intercostal space. The length of the access incision was observed to have no significant effect on the rates of procedure-related complications. In international literature, there are two types of VATS procedures distinguished: c-VATS (complete VATS) and a-VATS (assisted VATS). In completely video-assisted thoracoscopic surgery (cVATS), the procedure is done under control of a video camera and with instruments inserted through the thoracoscopic access ports. In assisted video-assisted thoracoscopic surgery (aVATS), the procedure is performed through video-assisted minithoracotomy [5]. The development of both c-VATS and a-VATS has been closely linked with technological advancements. State-of-the-art high-definition visualization, including 3D techniques, and the continuous refinement of instruments, with a focus on endostaplers with rotational member, promote the development of VATS lobectomy and its widespread introduction in clinical practice. According to STS (STS-GTD, Society of Thoracic Surgeons General Thoracic Database) data, the percentage of VATS lobectomy procedures has increased progressively as compared to traditional open approach surgeries, from 10% in 2002 to 29% in 2007 [6]. In 2014, the database included records of 56,656 patients who underwent VATS [7, 8]. The highest percentage of VATS lobectomy versus thoracotomy procedures was reported for Denmark (55%). In Copenhagen, VATS lobectomy was used in 80% of patients [9]. The percentage rates of VATS lobectomy versus open approach surgeries increased from 2% in 1993 to 14% in 2011 in UK and Ireland [10]. In Poland, the KRRP registry (national lung cancer registry) includes data of 305 VATS, accounting for 10.8% of lobectomies in patients with stage I and II nonsmall cell lung cancer. VATS lobectomy is defined as

- 4–6 cm incision made between the ribs,
- no rib spreading,
- procedure performed under control of VATS camera.

## 3. Indications and Contraindications

Indications for VATS lobectomy remain controversial, even more so as the traditional open chest surgery is a broadly recognised and well-established approach. VATS is generally recognised as a modality dedicated to the management of early stage cancer (stages I and II) with no signs of lymph node invasion [11]. Pulmonary function values are an important eligibility criterion; that is, single-lung ventilation is considered mandatory for VATS lobectomy. However, VATS lobectomy procedures in patients with predicted postoperative FEV1 < 30% have been reported as well. Although some authors suggest that in this group of patient VATS- segmentectomy is more favourable. Zhong et al. [12] presented that VATS-segmentectomy is a safe option and provides comparable oncologic results to VATS-lobectomy specially in stage IA nonsmall cell lung cancer. The essential condition is to carefully qualify patients based on the findings of imaging procedures and invasive diagnostic tests (bronchoscopy, EBUS-TBNA, EUS-FNA, and mediastinoscopy). The dynamic development of diagnostic imaging techniques has been inextricably linked with the increasingly common application of VATS in clinical practice. For example, local stage of tumour progression can be now examined with PET-CT scanning. However, sceptics argue that these diagnostic procedures have considerable limitations. Herth et al. [13] reported that EBUS-TBNA was detected as much as 19% of lymph node involvement in a group of 100 patients with <10 mm lymph nodes in CT and absence of metabolically active lesions in PET-CT. This is particularly important in the context of VATS lobectomy since lymph node involvement is considered one of contraindications to VATS. Still, with the increasing experience and technical advancement, the eligibility criteria for VATS lobectomy have been progressively extended to include more advanced stages of cancer. According to the previous updates in eligibility criteria, patients with >6 cm or T3 tumours are considered ineligible for VATS lobectomy [14]. Here is a list of relative contraindications:

- dense pleural adhesions (especially for less experienced surgeons);
- tumours visible by bronchoscopy (where the lesion is directly adjacent to the origin of the lobe and a possible sleeve resection might be needed);
- lymphadenopathy (related to a benign tumour or the underlying condition);
- preoperative radiation therapy or chemotherapy;
- tumour infiltration to the chest wall.

## 4. Conversion to Thoracotomy and VATS Complications

An open thoracotomy remains the gold standard approach to thoracic procedures in lung cancer and should be considered whenever individual surgeon's experience is too limited for a safe and effective VATS lobectomy, or if any life-threatening perioperative complications emerge. The causes of intraoperative conversions to thoracotomy during VATS lobectomy can be divided into the following groups: perioperative complications, technical reasons, anatomy, and tumour related causes. In clinical studies, nearly 30% of all conversions were not related to the tumour [15]. In a study by Krasna et al. [15], 37% of conversions were due to bleeding, 30% for local advancement, 23% for dense pleural adhesions, 7% for technical problems with the stapler, and 3% for pneumothorax on the opposite side (3%).

The percentage of complications following VATS lobectomy varies from 6% to 34.2%, estimated on a large volume of clinical data [16–18]. The risk of complications in traditional open thoracotomy can be as high as 58% [16]. Whitson et al. [17] reported the following common postoperative complications: prolonged air leak of >7 days (56%), atrial fibrillation (32%), massive pleural drainage (14%), pneumonia (13%), and myocardial infarction (10%). Bronchopleural fistula was present in 3% of patients. In studies by Sakuraba et al. [19], the rates of atelectasis requiring bronchoscopy were lower in VATS group as compared to patients undergoing open thoracotomy (0% versus 6.3%, resp.). Moreover, chest tube drainage >7 days (1.5 versus 10.8%, resp.) and hospital length of stay (5 versus 7 days, resp.) scores were better in VATS versus open.

## 5. Benefits of VATS versus Open Thoracotomy

Major benefits of VATS relate to reduced pain following surgery. Pain was demonstrated to occur in up to 50–70% of patients at two months or more after thoracotomy procedures using a retractor, and over 40% of patients may still have some degree of pain at one year after surgery, with 5% of patients experiencing significant levels of pain. Pain can cause a number of peri- and postoperative complications both immediately and long after the surgery [19]. Less pain, reduced chest drain durations, and shorter lengths of stay and recovery period are highlighted as the main advantages of VATS. In a study by Sakuraba et al. [19], statistically significant differences were demonstrated in 752 patients who underwent either video-assisted thoracoscopic or open lobectomy: shorter median operative time (video-assisted thoracoscopy 117.5 minutes versus open 171.5 minutes), lower chest tubes drainage (987 mL in video-assisted thoracoscopy versus 1504 mL in open lobectomy), and shorter length of stay (4.5 days versus 7 days). A statistically significant difference was also found in perioperative blood loss to the advantage of VATS.

There is no objective method to measure pain intensity, and the perception of pain intensity is difficult to analyse. Interesting to note is that according to some reports there are no significant differences between the intensity and duration of pain following VATS lobectomy and open thoracotomy. In a study by Scott et al. [20] from the renown Memorial Sloan Kettering Cancer Center, the percentage of patients experiencing intensive postoperative pain at 4, 8, and 12 months after VATS and open thoracotomy was comparable (14%, 16%, 14%, and 10%,7 for VATS and 11%, 23%, 18%, 12%, and 6% for open thoracotomy). Postoperative respiratory parameters (FEV1 and FVC) were demonstrated to be significantly higher after VATS as compared to open thoracotomy in a number of studies [17]. The latest studies investigate the patterns of postoperative immunosuppression. VATS was associated with a less significant reduction in lymphocyte T (CD4), CRP, and interleukin 6 counts. These data may be indicative of a lower degree of invasiveness of VATS, an important precondition for shorter postsurgery recovery and recuperation period [21]. However, better immune system parameters do not translate directly into lower risk of postoperational infection complications in patients after VATS versus open thoracotomy.

## 6. Oncologic Aspect

Oncologic aspect of video-assisted thoracic surgery remains controversial. Sceptics argue that the decreased invasiveness of VATS affects the radicality of tumour resection, which translates into poorer long-term outcomes of cancer treatment as compared to open thoracotomy. On the other hand, those in favour of VATS lobectomy claim that the principles of surgical treatment remain unaffected by the use of a different surgical access technique. The first step is to assess patient eligibility for VATS lobectomy as this technique should be essentially used in patients with early stage lung cancer. Verifying patient eligibility should preferably involve all diagnostic methods (diagnostic imaging techniques: CT, PET-CT, and NMR and endoscopic methods bronchoscopy, EBUS-TBNA, and EUS-FNA) and in cases of doubt, invasive methods as well (mediastinoscopy). The learning curve is another important factor. Many studies on VATS lobectomy include a separate analysis of VATS lobectomy in early and late phase of a surgeon's learning curve. A lot of attention is devoted to the correct evaluation of the condition of lymph nodes and changes in the mediastinum. In one of the latest studies, the findings seem to support the arguments in favour of open thoracotomy [22]. The mean number of nodes dissected in the VATS group was significantly lower (9.9/patient) as compared to the open group (14.7/patient, *p* value 0.003). Particularly significant differences were reported for N2 group: 4.7 and 8.5/patient in VATS and open groups, respectively (*p* value 0.002). The differences were insignificant in N1 group. In the open lobectomy group, 24.6% of patients were upstaged from N0 to N1 and from N1 to N2 compared with 10% in the VATS group. These findings may fuel scepticism for VATS. However, the 3-year survival was similar between the groups (89.9% for VATS versus 84.7% for open lobectomy). Also, comparable effectiveness of both methods has been reported in a number of papers. Merritt et al. [23] demonstrated that the number of lymph nodes removed per patient was 24 and 25.1 in VATS on the left and the right side, respectively, and was comparable to the number of lymph nodes removed in an open chest procedure: 21.1 and 25.2, respectively. These are the findings of a prospective and randomized study. In a study by Whitson et al. [17], 5-year survival rate in VATS patients of 75% was comparable to lobectomy with thoracotomy. In Palade et al. [24], the 5-year overall survival rate in the VATS patients of 95% was higher than in the open group. Another issue related to VATS is the rate of recurrences following videothoracoscopic treatment. Of particular concern are local recurrences and minithoracotomy site recurrences associated with the very narrow access space. Walker et al. [25] demonstrated a lower recurrence rate in VATS versus open thoracotomy group of 18% and 29%, respectively. In patients after thoracotomy, the percentage of distant metastases was higher (63% versus 32% in VATS). Surprising was the fact that the percentage of synchronous primary tumours confirmed perioperatively in the open group was higher than in VATS (12% versus 7%, resp.). The risk of minithoracotomy site recurrences could not be confirmed in a number of studies, which is likely to result from the current prevention measures (surgical field protection and retrieval bags).

## 7. Learning Curve

VATS lobectomy is a relatively young technique and is still evolving. The majority of thoracic surgeons are extensively trained in traditional open chest surgery. Training in traditional thoracic surgery is the essential precondition for later training in VATS and makes surgeons ready for emergency conversion to open thoracotomy. Obligatory education in VATS lobectomy is not broadly used and is only beginning to emerge in Poland. Being skillful in anatomical pulmonary resection accompanied by thoracotomy for transthoracic access does not necessarily translate into the ability to perform VATS. This is possibly due to the specific visualization of the operating field (highly enlarged 2D images) and the use of different instruments. Effective identification of anatomic structures may be difficult in the initial phase of the learning curve. Flores et al. [26] compared different surgical aspects of VATS in the first 20 patients (group A) and patients operated in late phase of the surgeon's learning curve (group B). The study covered all VATS lobectomies performed by a single experienced thoracic surgeon over a 3-year period. The conversion rate was 25% in group A and only 5% in group B. The median operative time was significantly shorter in group B (150 min versus 192.5 min in group A). Initially, 25% of all lobectomies were performed with the VATS technique, which has increased to 75% in late stage of the learning curve. The authors argue that a surgeon can acquire the VATS lobectomy technique with minimum 20 cases. Other authors claim that surgeons have to overcome a learning curve of at least 25 cases and previous 100 cases of “smaller” VATS procedures [27]. Patient selection is particularly important in early stage of the learning curve. Small peripheral lesions are preferred instead of central tumours. Inexperienced surgeons should avoid cases with noncancer lymphadenopathy. The choice of the resected lobe is also important. Lower lobe resection is considered easier due to the limited number of blood vessels.

## 8. New Trends

Minimally invasive pulmonary resection techniques have been evolving to achieve improved radicality and to significantly reduce perioperative injury. A single-port or “uniport” access performed with only one incision has been described in a number of recent papers [28]. If used for diagnostic or limited resection purposes, the uniport technique and traditional videothoracoscopy proved comparable in terms of effectiveness. Wang et al. [29] investigated the uniport VATS surgery in patients with early stage cancer (group A) and T3 or T4 tumors (group B). The conversion rate was significantly lower in group A than in group B (1.1 versus 6.5%). Surgical time was longer (144 versus 183 minutes) in group B. The majority of patients from both groups did not experience any significant pain (82.8% in group A and 86% in group B). 65.5% of patients in group A were discharged in the first 72 hours versus 51.2% of patients in group B. The 30-month survival was 90.4% for group A and 73.7% for group B. Based on the above findings, it can be concluded that the uniportal VATS lobectomy will be soon broadly used in clinical practice. The progress in VATS lobectomy is closely linked with the development of new instruments. Needlescopic video-assisted thoracic surgery is where traditional intercostal ports are avoided. Another trend is robot-assisted minimally invasive techniques. Gonzalez-Rivas et al. [30] performed a series of robot-assisted approaches to lung cancer resection in 54 patients using a four-arm Vinci Robotic System. The conversion rate was 13%. Postoperative complications were observed in 20% of patients. The median number of lymph nodes removed was compared in the 2 groups (17.5 versus 17/patient). Postoperative hospitalization was significantly shorter after robotic than after open operations (4.5 versus 6 days). However, the cost of four-arm robotic lobectomy may be considered a significant disadvantage. Robot-assisted resection is by around EUR 2,000 more expensive than VATS lobectomy and thoracotomy. New developments also emerge in anaesthesia for thoracic surgery to limit side effects. Thoracoscopic surgery with regional anaesthesia and without endotracheal intubation was described in a number of papers. Lung collapse occurs naturally on introduction of the first port to the pleura. In a study by Veronesi et al. [31], 446 patients were treated by nonintubated thoracoscopic surgery, with lobectomy performed in 189 patients. 3.6% of patients required conversion to thoracotomy. Anaesthetic side effects were noted in 6.3% of patients; 3.6% of patients required conversion to tracheal intubation. Nonintubated thoracoscopic surgery may be considered in patients with decreased respiratory reserve, at risk of serious perioperative complications associated with traditional anaesthesia and one lung ventilation.

## 9. Summary

The evolution of VATS lobectomy is driven by technological advancement and refinement of surgical instruments. It is increasingly used worldwide. In some medical centres, this approach has become the dominant method of lung cancer surgery. The effectiveness of VATS lobectomy is a controversial matter. The only way to address these controversies is to perform large-scale clinical studies covering large population of patients. The eligibility criteria for VATS lobectomy have been progressively extending to accommodate advanced stages of cancer. Proper patient selection is considered of paramount importance, also in terms of surgeon's experience. Research shows that minimally invasive treatment methods are as effective as traditional methods but are accompanied by less suffering. In the coming years, we will perhaps be able to objectively assess the benefits of VATS by learning more about this thoracic surgery technique.
